# The Gillingham Case

**Published:** 1903-04-04

**Authors:** 


### The Hospital.

A Journal of

The Medical Sciences and Hospital Administration.

Vol. XXXIV.—No. 862. | Edited by Sir Henry Burdett, K.C.B., and Solomon C. Smith, M.D., M.R.C.P. | [April 4, 1903.

LIBRARY SURGEON GENERAL'S OFFICE APR. 13. 1904 185205

### The Gillingham Case.

The case of Chapman and wife against the Gillingham Urban District Council, which occupied Mr. Justice Grantham, an array of counsel, and a special jury, for four days last week, is one replete with warnings alike to all local sanitary authorities and to those who have the responsibility of advising them. It appeared from the evidence that the plaintiff, Chapman, was a market gardener within the Gillingham district, occupying a house and land for the purposes of his business. In December 1901, a case of small-pox occurred in a crowded part of the district, and the patient, a man, was in the first place removed to a tent erected in the grounds of the infectious hospital belonging to the defendants, and fifty or sixty yards from the building, which was occupied at the time by several patients with maladies other than small-pox. Whether or not it was believed that these patients would be more susceptible to contagion than healthy persons does not appear; but, at any rate, it was felt that the position was an improper one, and a member of the defendant council offered a vacant stable belonging to him as a temporary hospital for the case. The stable had been built to be an appendage to an intended residence, and it was situate about 200 yards from Chapman's house, and within 37 feet of his land. The patient was transferred to the stable on December 5th, and was placed under the care of a male nurse, formerly a hospital sergeant in the army, who was instructed to use due care in the disinfection and disposal of all excreta and other refuse, but who, it was alleged, had been negligent in the discharge of this portion of his duty. At any rate, on December 18th Chapman's little daughter, eight years old, who had not been outside her father's land for six weeks before the arrival of the case of small-pox, developed the disease, and died on December 29th. The plaintiff and his wife also contracted the disease and recovered, but his father and mother and his wife's mother, who were resident close by, the last next door to him, all died of it. The action was brought to recover damages for the general loss and injury sustained; and it was alleged on the part of the plaintiff that the defendants acted negligently in bringing a source of infection so close to his premises. The argument was that the little girl contracted the disease from the vicinity of the temporary "hospital," to which a second patient was brought after a time, while a third one was placed in a caravan close by; and that, even if the adults contracted the disease from the child, the source of the whole mischief was traceable to the original wrongful act of the defendants. It was admitted that the defendants were justified in removing the case of small-pox from the crowded locality in which it had appeared, and also that they made haste to supersede their temporary arrangements by the establishment of a proper hospital for small-pox at Wigmore, but it was urged that they had not exercised due care in the selection of the temporary receiving house, and that to this fault all the sufferings of the plaintiff and his relatives had been due. The jury found that due caution had not been exercised, and awarded £250 damages to the plaintiff, for which amount judgment was given and a stay of execution refused.

It appeared in the course of the case that the little girl who died had not been vaccinated, but nothing was said as to the vaccination of the other persons who contracted the disease. Chapman had other children, but they were sent away as soon as the nature of the case became apparent, and seem to have escaped. The medical witnesses called on either side were pressed as to the aerial conveyance of infection, with especial reference to the recent report of the Local Government Board touching the epidemic at Purfleet, and some of them were bold enough to express an opinion that such conveyance did not occur, and that small-pox was only communicated by direct contact with infected persons or things. The only really important piece of medical evidence was that of Dr. Ricketts, the medical superintendent of the small-pox hospital of the Metropolitan Asylums Board, who said that in his hospital, last year, there were 700 people, nurses, scrubbers, and others, employed on the premises. All of them, with one exception, had been recently vaccinated, and the exception was the only one of the whole number who caught small-pox. The evidence as to the alleged negligence of the nurse in dealing with excreta was conflicting; and the whole case may be said to have turned upon the unsuitability of the position of the stable, in view of the proximity of the plaintiff's land and dwelling house. Thanks to the Anti-Vaccination League, or whatever its name may be, small-pox, for

185205

some years to come at least, is likely to be always with us; and it is therefore necessary to be prepared against its incidence. But, inasmuch as it is a malady against which every person may protect himself, and every parent his children, we think that its occurrence should form no ground for actions at law against either local authorities or private persons. The Government will, it may be presumed, introduce legislation on the subject next year; and it would be perfectly fair to enact that no damages should in any case be recoverable in a court of law, on account of the occurrence of small-pox in any persons, who, having passed the statutory age, still remain unvaccinated.

